# Little lives—reading between the lines: insights from the Northampton Infirmary Eighteenth Century Child Admission Database

**DOI:** 10.1017/mdh.2025.10026

**Published:** 2025-07

**Authors:** Madeleine Mant, Judy Chau, Bryce Hull, Maryam Khan, Mollie Sheptenko, Mia Taranissi, Charlotte Parry, Fred O’Dell, Andrew Williams

**Affiliations:** 1 Department of Anthropology, University of Toronto Mississauga, Mississauga, ON, Canada; 2 Department of Anthropology, University of Toronto, Toronto, ON, Canada; 3 Department of English, University of Toronto Scarborough, Scarborough, ON, Canada; 4 Northampton General Hospital, Museum and Archive, Northampton, United Kingdom; 5 Division of Life Sciences, Faculty of Arts, Science and Technology, University of Northampton, Northampton, United Kingdom

**Keywords:** morbidity, paediatrics, infectious disease, trauma, skin disease, hospital child care

## Abstract

The presence of children in eighteenth-century English voluntary hospitals is an area of increasing interest and attention. The Northampton Infirmary admission records detail inpatient and outpatient ages from 1744 to 1804, allowing for longitudinal investigations of children in the institution. The most common distempers affecting children were surgical infections, infectious diseases, and skin diseases; fifty-six per cent of the child patients were male and 43.3 per cent were female. Nearly seventy-five per cent of children left the hospital ‘cured’. This article outlines the Northampton Infirmary Eighteenth Century Child Admission Database, and demonstrates how the patterning of distempers within and among children provides insight into the health journeys of eighteenth-century children through the lens of their bodies, their parents, and their institutional recommenders.

## Introduction

Recently, the generality espoused by Fielding Garrison that ‘Pediatrics as a specialised branch of medicine had no real existence before the middle of the nineteenth century…’^1^ has come under scrutiny. Eighteenth-century medical texts such as Cadogan’s *An essay upon nursing and management of children* (1748), Buchan’s *Domestic Medicine* (1769), Armstrong’s *Essay on the diseases most fatal to infants* (1767), and Underwood’s *A treatise on the diseases of children with directions for the management of infants from the birth* (1784) indicate the very real existence of care for children before the founding of specialised institutions such as The Hospital for Sick Children, Great Ormond Street in London in 1852.^2^ Indeed, Thomas Phaer’s *The Boke of Chyldren* (1544) is the first English text regarding the disease of children, indicating a longstanding awareness and interest in the ailments of the young.^3^ Hannah Newton, in her studies of early modern sick children, introduced the term ‘children’s physic’, to refer to the specialised care provided to children, which Claire Rennie argues can be extended to the end of the eighteenth century.^4^ While institutions such as the London Foundling Hospital (founded 1745) and the Dispensary for the Infant Poor (1769–1781) provided local care for children,^5^ a broader understanding of English children’s interactions with institutional healthcare must be drawn from the voluntary hospitals. Children were, in fact, accepted as patients at voluntary eighteenth-century hospitals in contravention of the official rules of the institutions.^6^ Driven by the Enlightenment, the voluntary hospital movement resulted in the founding of thirty-five voluntary hospitals in England and seven in Scotland over the eighteenth century.^7^ Such hospitals were dependent upon donations from subscribers and served those unable to afford private physician visits, with the expectation that individuals would return to work following their care.

Digging into the history of child patients ‘from below’, as encouraged by Roy Porter, follows the concept that ‘…managing and treating sickness remained very largely in the hands of the sufferers themselves and their circles…’ and ‘…if we cast our nets more widely, a rich haul of materials will tell us about the communal minds and hearts of the sick…’.^8^ Even so, this requires the survival and curation of patient records that record in- and outpatient ages. The survival of such eighteenth-century hospital admission records is rare enough; those with age recorded are even rarer. This makes the Admission Register of the Northampton General Infirmary a particularly important document in the study of child patients. Age is recorded from day one; in fact, the first patient admitted was a child, Thomasin Grace.^9^ This admission is important, as the voluntary hospital statutes make clear the intent of these institutions:

That no woman big with child, no Children under *seven* Years of Age, (except as in the *foregoing* Rule) no Persons disorder’d in their Senses, or suspected to have the *Smallpox, Itch*, or other *infectious* Distemper: nor any who are apprehended to be in a *consumptive* or dying Condition, or who are supposed to have *Venereal Disease*, be admitted into the Hospital as in-*Patients*, or any Account whatever, or permitted to stay in it.^10^

Children were thought to be particularly infectious, disruptive to other patients, and generally hard to treat, and therefore not the focus of voluntary hospitals’ limited resources, which sought to address cases deemed ‘curable’ and get working-class people back to work.^11^ Despite these limitations, children were treated in voluntary hospitals; Levene and colleagues note that ‘…observing the rules on childcare more in their breach than their observance…’ is revelatory regarding conceptions of children during the eighteenth century.^12^ In their comparative study of two years of admission records from five provincial English hospitals—Bristol Royal Infirmary, Northampton General Hospital, Royal Victoria Hospital, Newcastle, Manchester Royal Infirmary, and Chester Royal Infirmary—Levene and colleagues found that children (thirteen and under) were treated at all five hospitals. On average, about thirteen per cent of the patients seen were children, and about sixty per cent of the children were male.^13^ The authors trace the pathways of care these provincial children likely received, placing the hospital within the wider nexus of family and community care. This comparative dataset has also yielded a further exploration of neurodisability; the authors highlight how the quantitative data provide useful material to put in dialogue with contemporary treatises such as Buchan’s *Domestic Medicine.* ^14^

Explorations of voluntary hospitals and their child patients underline what Claire Phillips has emphasised regarding working-class parents, namely that ‘…parents of sick children had a number of options available to them, and parents were aware of these options’, including voluntary hospitals, dispensaries, institutions focused upon children such as the Foundling Hospital, and domestic care.^15^ The relationships between these institutions and care for children varied widely, and not all had rules excluding children. For instance, Alun Withey’s exploration of the Bamburgh Castle Dispensary demonstrates that children were not only assessed at the institution, but there was also an organised smallpox inoculation program requiring them to present at the Castle.^16^ Withey underlines that eighteenth-century healthcare institutions likely overlapped in their types and manifestations of care more than has been previously recognised—relying on too narrow and distinct a definition of ‘hospital’ or ‘dispensary’ may underestimate the complexity and type of care being provided.^17^ Certainly, the Northampton General Infirmary was treating both in- and outpatients and, in fact, also provided smallpox inoculation from 1804.^18^

While two years of records from the Northampton General Infirmary were investigated in the above studies, the full admission register for the years in which age is recorded (1744–1804) has now been digitised and transcribed, forming the basis of the Northampton Infirmary Eighteenth Century Child Admission Database (NIECCAD). Investigation of this new database allows for a diachronic, sustained, and more nuanced picture of children’s interactions with healthcare in an English town. These records were examined to discover which distempers most commonly affected child patients over a sixty-year span, how long they stayed in the hospital, and with what outcomes. This resulted in an investigation of 4,163 child admissions, comprised of fifty-six per cent male and 43.3 per cent female children. Despite the slightly higher proportion of male children assessed, there was little sex-based difference for most distempers. The fact that the infirmary records indicate how long a child was sick before presenting to the hospital allows for consideration of both domestic and institutional care in the healthcare encounters of the children of Northampton. The story of healthcare for Northampton children can certainly be considered ‘from below’, by examining the network of relationships involved in seeking care, including the child themself, their parent(s), and the hospital subscriber/recommender.

In this paper, we seek to investigate child morbidity, following the directive of Margaret Pelling, who noted that ‘…the study of morbidity, however difficult, can bring us close to conditions of life and daily experience, and even to the point of view of the object of study, in this case the child.’^19^ Before, however, exploring the relationship between the institution and the thousands of children who passed through its doors, it is useful to examine the role of Dr James Stonhouse, the physician who saw the need for such an institution in Northampton.

## Northampton General Infirmary background

As an undergraduate at St. John’s College, Oxford, James Stonhouse published a tract on deism that went to three editions (later in life, he attempted to retrieve and destroy all copies). Stonhouse then moved to London to commence his medical education under Dr Nicholls, a celebrated anatomist. Following this education at St. Thomas’ Hospital, London, from 1738 to 1740, he travelled to France to seek medical practice, but was soon back in England, settling briefly in Coventry.^20^ In April 1743, Dr James Stonhouse made the short journey to Northampton. The town had been recently rebuilt following a late-seventeenth-century fire, and Stonhouse saw the need therein and decided to stay. He advocated the necessity of Northampton having a county hospital for the sick and poor.^21^ Stonhouse originally had a reputation of being ‘…a most abandoned rake and an audacious deist’.^22^ However, he heard the Reverend Dr Philip Doddridge preaching a sermon at a funeral which so impressed him that he underwent a profound religious conversion; indeed, he later took up holy orders himself and published extensively on religious matters.^23^ Stonhouse and Doddridge became firm friends.

Stonhouse printed and circulated papers advocating for an infirmary and laying out the financial requirements. A subscription list was opened on 21 July 1743 to raise money for the purchase of a house for the purpose of such an infirmary. Doddridge preached a sermon on 4 September 1743 in Northampton with the aim of encouraging the town’s parishioners to contribute to the cause. This sermon was entitled ‘Compassion to the Sick recommended and urged, in favour of a design then opening to erect a County Infirmary there [Northampton] for the Relief of the Poor Sick and Lame.’ He said, ‘…I am about to lay before you in Favour of the Scheme, which is now opening upon us, for a County-Infirmary to be erected here…as I have great reason to hope it will; considering how noble a Charity it suggests, and how ready I have ever found you to comply with every Call of Providence to contribute liberally for the Assistance of the Necessitous.’^24^ Notably, in the preface, Doddridge gave the only two plausible objections to why an infirmary should not be built:

‘*That the distant* Parts of the County *can expect* little Benefit *by it;*—*and that any* private House, *which can be taken for the Purposes of a COUNTY HOSPITAL*, *can bear but* little Proportion *to what the* Necessities of so large a County *will require. But I hope, neither of these Objections will be found unanswerable; and if every Objector will do his Part towards removing them, I am sure they cannot be found so.’* ^25^

Sufficient funds were raised to obtain this objective, and a large townhouse was rented at £30 a year. The Northampton Infirmary came into being at an inaugural meeting on 20 September 1743, held at the Red Lion Inn in Northampton. *Statutes, Rules, and Orders for the Government of the County Hospital, For Sick and Lame Poor, Established in the Town of Northampton* was published in 1743, setting out the means of operation for the infirmary.^26^

**Figure 1.**
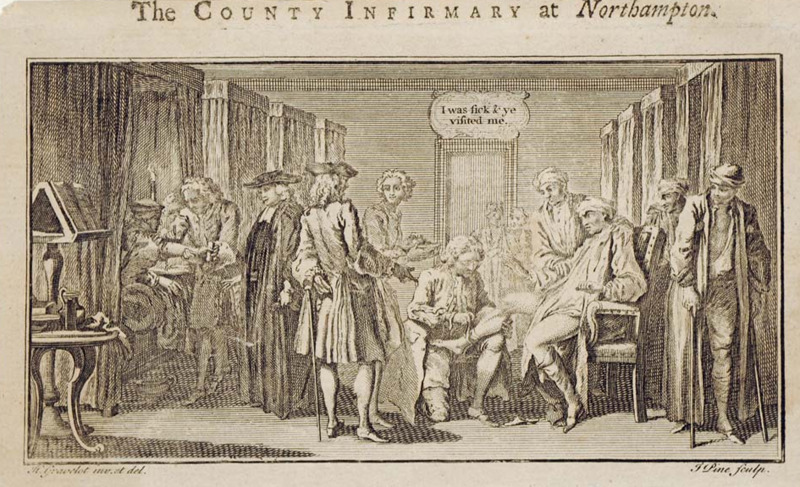
Illustration of the County Infirmary at Northampton. Originally printed in *The encouragement to works of charity and mercy, from Christ’s acceptance of them as done to himself. A sermon preached in the Parish Church of All Saints in Northampton, before the President and Governors of the County Infirmary for sick and lame poor on Thursday, March 29, 1744, by Richard Grey, D.D*. Source: Northampton General Hospital Archive (Permission for use provided kindly by the Northampton General Hospital Archive)

The eighteenth century saw English voluntary hospital numbers increase; indeed, the Northampton Infirmary was the sixth such provincial hospital in England.^27^ The Infirmary opened on 29 March 1744, and on that day, the first inpatients were admitted. A sermon of thankfulness was preached by Richard Grey, Rector of Hinton in Northamptonshire. He said, ‘The Relief, which the Miserable here meet with, must naturally lead them to a due sense, and thankful Acknowledgement of God’s Goodness and Mercy, in raising up Benefactors to give them Help in time of need…’^28^ In fact, there is a rule to that effect: Rule twelve of Rules for the Admission of Patients states, ‘That when the Patients are cured, they be enjoined by the Chairman to return Thanks, in their respective Places of Worship.’^29^ This sermon was published in 1744; Figure 1 appeared in this volume, visually demonstrating the Infirmary in therapeutic action. Such an image alongside the written word would have been used to raise awareness, secure the Infirmary’s good reputation, and ideally assist in maintaining a sustained income stream for the institution. These religious overtones are further emphasised by Dr Stonhouse in *Friendly Advice to a Patient*, where he outlines that:

The first necessary *Advice* will arise from the Consideration, that you are now under the afflicting Hand of God.—The Place in which this finds you, as a Patient, supposes two v*ery grievous* Afflictions concur; *namely*, That you are under some Illness, or unhappy Accident; and that you are so poor, as not to be able, at your own Expense, to procure *proper* Relief.—The Governors would not have admitted you, if they had not been persuaded, that this *was* your Case; And there would be so much Injustice and Wickedness in *deceiving* them into such a Persuasion, that I shall not entertain any such Supposition.^30^

The Infirmary at opening had thirty beds; this increased to sixty beds by 1773, and seventy beds by 1785. In the eighteenth century, admission was not so dependent on medical need but rather on a Governor’s or subscriber’s recommendation.^31^ In 1744, Northampton Infirmary treated 103 inpatients and seventy-nine outpatients.^32^ This increased the following year to 173 inpatients and 176 outpatients.^33^ By 1787, over 23,560 patients had been cured or benefited from their medical treatment since the Infirmary opened.^34^ From the late 1780s, it was apparent that the infirmary was not large enough to accommodate the increasing numbers of patients needing to be medically treated and a new site for a larger infirmary was sought. In September 1793, a new hospital opened on Northampton Fields to the east of town with a bed complement of 106 beds.^35^ By 1844, a century after it opened, the infirmary had treated over 114,000, mostly local, patients.^36^

## Northampton records

This manuscript considers the admission and discharge registers of the Northampton Infirmary, curated at the Northampton General Hospital Museum and Archive. Ages are recorded for inpatients from 1744 to 1804, while the reason for admission (distemper) is recorded from 1744 to 1801. The years 1797 and 1798 are missing. The registers variably include the patients’ name, age, whether they were made inpatient or outpatient, dates of admission and discharge, parish of origin, distemper, recommender, treating physician, outcome (‘how discharged’), and how long an individual had been ill with their distemper prior to admission. Recording of both age and distemper is excellent (>90 to 100 per cent complete) from 1744 to 1772. From 1773 onward, the recording of ages is less consistent, with completeness ranging from fifty-seven to eighty-two per cent until 1799, when the majority of the records have neither age nor distemper recorded. Despite this gradual downturn in data quality, here we present all pooled distemper results from 1744 to 1801 to ensure the greatest number of child inpatients are represented and investigated. The temporal trends and inpatient/outpatient results, however, are investigated with emphasis upon the 1744 to 1772 period.

Patients were separated into two groups: children seven and under, and children thirteen and under, to allow for meaningful comparisons between sites (Table 1).^37^ This differentiation is important as the Northampton Infirmary statutes, aligning with other English voluntary hospitals and institutions in Europe, explicitly outlawed the admittance of children under seven, except for a few exceptions.^38^

**Table 1.** Classification of diseases among child patients admitted to the Northampton Infirmary (1744-1801) in ranked order

Category of Admission	N (% of total child admissions)	Average length of stay in days (median)
Surgical Infection	571 (13.7)	101 (71)
Infectious Disease	519 (12.5)	82.5 (57)
Skin Disease	511 (12.3)	114.8 (85)
Musculoskeletal	403 (9.7)	113.2 (85)
Digestive System	366 (8.8)	98.8 (71)
Trauma	347 (8.3)	64.8 (43)
Eye Problems	233 (5.6)	105.9 (78)
Miscellaneous Surgical	228 (5.5)	143.7 (106)
Neurological	142 (3.4)	112.5 (74.5)
Miscellaneous Medical	125 (3.0)	76.4 (57)
Tumours, Cancers, Growths	119 (2.9)	101 (64)
Respiratory	90 (2.2)	105 (71)
Circulatory	40 (1.0)	111.9 (64)
Genitourinary	38 (0.9)	73.3 (53)
Surgical Procedure	1 (0.02)	211 (211)
Unknown	430 (10.3)	83.6 (71)

The total number of admissions of child patients (thirteen and under) treated at Northampton from 1744 to 1801 is 4,163; of these, 1,908 were seven and under. Fifty-six per cent of the sample were male children, 43.3 per cent were female children; it was not possible to determine the sex in 0.7 per cent of cases. In the years with consistent age recording (1744–1772), the range of patients under the age of seven ranges from 3.8 to 9.8 per cent of the total patients, while those thirteen and under range from 8.5 to 16.4 per cent of the total patients. These results indicate that young patients receiving both in- and outpatient treatment would not have been a rare sight at the Northampton Infirmary. These results align with those reported by Levene and colleagues, who found the proportion of patients aged seven and under to range from 1.5 to 15 per cent (average 6.4) and those thirteen and under to range from 6.1 to 24.8 per cent (average 13.3).^39^

The Northampton Infirmary was founded to serve the local parishes, and the admission records demonstrate this was the case: 97.3 per cent of recorded cases were from Northampton itself or Northamptonshire. This local context allows for an exploration of the risks facing children in mid-eighteenth-century Northampton and Northamptonshire. The environment and social context of poor children may be read between the lines of the admissions and discharges.

## Distempers

Table 1 outlines the reasons for children’s admissions to the Northampton Infirmary (1744–1801) using the diagnostic categories outlined by Risse in his investigation of the contemporary Royal Infirmary of Edinburgh.^40^ The recorded outcomes of the hospital visits are presented in Table 2. Children arrived at the Northampton Infirmary for in- and outpatient treatment for a vast array of conditions. From dropsy to worms, inflamed eyes to limb paralysis, whooping cough to skin eruptions, children were brought by their parents to seek care with myriad external and internal complaints.

**Table 2.** Result of hospital stay arranged in ranked order

Category of admission	Cured/relieved N (%)	Died N (%)	Made IP/OP N (%)	All other*	Total
Surgical infection	409 (71.6)	3 (0.5)	62 (10.9)	97 (17.0)	571
Infectious disease	377 (72.6)	6 (1.2)	8 (1.5)	128 (24.7)	519
Skin disease	379 (74.2)	4 (0.8)	30 (5.9)	98 (19.1)	511
Musculoskeletal	293 (72.7)	2 (0.5)	39 (9.7)	69 (17.1)	403
Digestive system	258 (70.5)	6 (1.6)	6 (1.6)	96 (26.2)	366
Trauma	266 (76.6)	3 (0.9)	8 (2.3)	70 (20.2)	347
Eye problems	171 (73.4)	2 (0.9)	18 (7.7)	42 (18.0)	233
Miscellaneous surgical	193 (84.6)	1 (0.4)	0	34 (14.9)	228
Neurological	88 (62.0)	0	13 (9.2)	41 (28.9)	142
Miscellaneous medical	101 (80.8)	0	8 (6.4)	16 (12.8)	125
Tumours, cancers, growths	103 (86.6)	0	7 (5.9)	9 (7.6)	119
Respiratory	58 (64.4)	3 (3.3)	3 (3.3)	26 (28.9)	90
Circulatory	22 (55.0)	4 (10.0)	3 (7.5)	11 (27.5)	40
Genitourinary	24 (63.2)	1 (2.6)	5 (13.2)	8 (21.1)	38
Surgical procedure	1 (100)	0	0	0	1
Unknown	360 (74.5)	3 (0.7)	24 (5.6)	43 (10.0)	430
Total	3103 (74.5)	38 (0.9)	234 (5.6)	788 (18.9)	4163

* All other = patients recorded as ‘ran away’, ‘incurable’, ‘own request’, or those marked ‘unknown’

As shown in Table 2, the majority (74.5 per cent) of child patients left the institution with a label of ‘Cured’ and less than one per cent died whilst in hospital. Hospital stays ranged widely; many patients stayed for a week, while others remained for months. The child mortality present in the hospital is low compared with other institutions, such as the contemporary London Foundling Hospital (records available from 1761–1771, 1777, 1787, 1797). Weekly records from the institution show that 11.5 per cent of those recorded died, mostly commonly of smallpox, measles, and general fevers; these deaths represent children in the institution about four to five years old (returned following wet nursing) and twelve to thirteen years old (awaiting apprenticeship).^41^ Similarly, the limited extant records of the London Hospital (1760, 1791, 1792) show that 9.3 per cent of the child inpatients died in hospital.^42^ Cherry has posited that patients deemed ‘incurable’ may have been discharged to avoid their inclusion in hospital mortality statistics, but notes also that provincial hospitals were generally honest in their reporting of years involving high mortality, so it is unlikely the statistics were significantly altered to save face.^43^ It is likely that children who died in Northampton did so at home rather than at the hospital. The children recorded as dying at Northampton most commonly died of fevers (e.g., rheumatic fever, low fever, intermitting fever) and digestive disease (e.g., bloody stools, vomiting, worms, diarrhoea). The two fever deaths for which ‘how long ill before admission’ was recorded were of only three weeks duration, whereas the digestive system deaths ranged from two months to a year duration.

Surgical infections, including abscesses, ulcers, boils, and swellings, were the most common reason for seeking care, making up 13.7 per cent of the total sample. Male children comprised 54.1 per cent of those with such complaints, with female children making up 44.8 per cent of the sample (1.1 per cent unknown sex). Such cases would be allowable as inpatients, and indeed 46.4 per cent of the sample were admitted as inpatients (1744–1774). Where ‘how long ill before admission’ was recorded (277 cases), most child patients (36.5 per cent) had been dealing with their condition for over a month, but less than a year. Nearly 30 per cent were recorded as being ill for over a year, while twenty-seven per cent were suffering for less than a month, and only 6.9 per cent were admitted as accidents or ‘a case admitting no delay.’ Treatments for these issues were straightforward according to Buchan; those with strong constitutions should be provided ‘…a slender diluting diet, plentiful bleeding and repeated purges.’^44^ If the inflammation should continue, the physician should ‘…promote the suppuration’ by means of poultices before opening the lesion with ‘…a lancet or by means of caustic.’^45^ Such interventions would result in obvious effects, explaining the 71.6 per cent success rate of leaving child patients cured/relieved of their symptoms. The boils, impostumes, and swellings affected the children of Northampton were not unique to the parish; in the Bristol Royal Infirmary (1756, 1779) and the Royal Victoria Hospital, Newcastle (1779), infirmaries’ surgical infections comprised 9.9 to 15.9 per cent of all child patient distempers.^46^

Infectious disease, which comprised 12.5 per cent of the total cases, was the second most populous category of distemper. Such a result would not have been a surprise to Underwood, who wrote that ‘Young children, however, are disposed to some febrile complaints peculiar to themselves…’, so much so that he ‘…enlarged this edition considerably, with the design of taking in all their complaints.’^47^ The majority of cases were various forms of fever, including ‘intermittent’, ‘slow’, ‘tertian’, ‘quotidian’, and ‘low’ varieties, aligning with the numerous feverish states described by Dobson from seventeenth and eighteenth-century Southeastern English hospital registers.^48^ Buchan also emphasised the infectiousness of children, cautioning that ‘…their breathing not only renders the place unwholesome, but, if any one of them happens to be diseased, the rest catch the infection…’^49^ The sex of children treated for infectious disease was evenly split between males and females. The admittance of child patients, indeed any patients, as inpatients with *infectious* distempers, was forbidden, and it is evident that the staff of Northampton took this rule seriously. Of the 213 patients from 1744 to 1773 for whom inpatient/outpatient status was recorded, only thirty-two (fifteen per cent) were made inpatients. The reasons for these case-by-case decisions are not immediately apparent, though they could be interpreted as particularly distressing cases. All are registered as originating in Northampton itself or a surrounding parish (except one child, noted as ‘Soldier’), with ages ranging from seven to thirteen. The children are recommended by myriad different recommenders, suggesting these inpatients are not the result of a single individual’s influence, unlike at Chester Royal Infirmary, where the notable number of children admitted for infectious disease may be attributed to the work of physician John Haygarth in building fever wards at the institution.^50^ For those children for whom the ‘how long before admission’ was recorded (n=233), 46.4 per cent had been ill less than a month, 43.8 per cent between a month and a year, and 9.9 per cent were ill for over a year.

Skin disease was nearly as populous a category as infectious disease, with 12.3 per cent of cases. The cases were essentially evenly split between male (49.8 per cent) and female (49.6 per cent) patients. This category included a range of acute and chronic issues, with some patients arriving at the hospital within days of their condition arising, and others listed as experiencing their condition ‘from birth.’ Children dealing with skin disease for whom the ‘how long ill before admission’ were recorded (n=314) fell mainly into the year+ category (55.4 per cent), with 30.9 per cent dealing with their illness from over a month to less than a year, 13.3 per cent for less than a month, and only one individual seen emergently. Similar to those deemed to have infectious distempers, those with skin disease (from 1744–1773) were mostly (seventy-three per cent) treated as outpatients. Further, those with ‘cutaneous eruptions’ and skin lesions attributed to leprosy and tuberculosis are seen as both inpatients and outpatients. Tinea capitis is described by Buchan as ‘The most obstinate of all the eruptions incident to children…’ and he suggests increasingly invasive steps to attempt a cure:

The cure ought always first to be attempted by keeping the head very clean, cutting off the hair, combing and brushing away the scabs, &c. If this be not sufficient, let the head be shaved once a-week, or oftener, and washed daily with soap-suds, or lime-water. Should these fail, a plaster of black pitch may be applied, in order to pull out the hair by the roots.^51^

Levene and Siena, discussing eighteenth-century reporting on pauper children’s illnesses, indicate that while infectious skin issues among children are frequently discussed, there is no blame being placed upon the children themselves, despite the framing of skin conditions as connected to sin and poverty.^52^ Contemporary texts, such as Spooner’s *A Short Account of the Itch*, even emphasise the ease of spread of conditions like the itch.^53^ The root of the issue, according to Buchan, is the lack of cleanliness often found amongst the poor, as he notes that ‘The children of the poor, and of all who despise cleanliness, are almost constantly found to swarm with vermin, and are generally covered with the scab, itch, and other eruptions.’^54^ Manchester Infirmary, in Levene and colleagues’ investigation, counted skin disease as the largest single category for child patients in 1756,^55^ with similar descriptions of ‘eruptions’ and ‘itch’ appearing alongside scrophulous and scorbutic skin lesions. Mathisen’s investigation of the London Foundling Hospital (1761–1797) similarly found that itch was the most common recorded condition.^56^

Digestive disease was mainly treated as an outpatient concern, with 86.4 per cent of cases treated in this manner. Cases were nearly even between male (47.5 per cent) and female children (51.1 per cent). Children presented with worms, diarrhoea, stomach pains, flux, and various bowel complaints, of duration ranging from less than a month (28.6 per cent), more than a month but less than a year (50.8 per cent), and 20.6 per cent cases for over a year. Cherry highlights the connection between crowded and unsanitary housing conditions influencing the appearance of digestive symptoms.^57^ Worms, often accompanied by fever and/or ague, were the most common type of digestive disease recorded. Buchan acknowledges that ‘Children are more liable to this disease than adults…’ and, concerningly, that ‘…no disease more frequently baffles the physician’s skill.’ Purgatives should be provided to expel the worms, after which parents are encouraged to allow their children ‘…plenty of exercise in the open air…’, avoid ‘…trashy fruits…’ and particularly to avoid the worm medicines shilled by quacks, which could contain sufficient mercury to cause poisoning.^58^ The high proportion of cases recorded as cured/relieved (70.5 per cent) may refer to initial stages of relief, as without a change in environmental conditions, it would be possible to suffer repeated bouts of worms and worm fever.

The musculoskeletal (fourth most populous) and traumatic (sixth most populous) categories of distemper resulted in the greatest sex disparity. The musculoskeletal category—comprising consultations for rheumatism, weakness, muscle contraction, and distortions of the body—comprised 63.5 per cent male children and 35.2 per cent female children. Trauma comprised 72.8 per cent male children and 26.6 per cent female children. Musculoskeletal cases were seen as inpatients in 45.7 per cent of cases and as outpatients in 54.3 per cent of cases; the trauma cases are more commonly outpatients (61.5 per cent of cases). Finally, approximately one quarter of musculoskeletal cases were seen less than a month after the onset of symptoms, 34.6 per cent between one and twelve months, and 39.8 per cent were cases of over a year in duration. Several are noted ‘from infancy’ or ‘from birth’, suggesting potential congenital conditions such as hip dysplasia. Records from the Foundling Hospital detail cases of children with various physical disabilities, including congenital limb differences. Ashley Mathisen, drawing upon these records, emphasises that childhood disability ‘…cannot be examined outside the context of apprenticeship and child labor…’ as such musculoskeletal conditions did not necessarily preclude children from taking on apprenticeships.^59^ In contrast, 83.2 per cent of trauma cases at Northampton were recorded as ‘Accident’, including injurious kicks from horses and falls, with another 10.9 per cent of cases recorded as being less than a month old.

Eye problems comprised 45.9 per cent of male children and 53.2 per cent of female children. This category included diagnoses of blindness from ulcers, bad eyes, ophthalmia (eye inflammation), and cataracts. Time from onset to hospital treatment was variable; 33.9 per cent of cases were seen in less than a month, 43.2 per cent between one and twelve months, and 22.9 per cent of cases were more than a year in duration, with two cases of cataracts of nearly eleven years in duration. Underwood differentiates between and offers treatment options for ophthalmia, leucoma, cataracts, gutta serena, and styes in children, noting that infants often have inflamed eyes immediately postpartum, which is not overly concerning. The appearance of ‘purulent ophthalmy’ is highlighted as a means for alarm, and leeches, blisters, and scarifications are noted as potential means of treatment.^60^ The Foundling Hospital’s description of eye disorders was similar to Northampton, in which practitioners, even when using labels like ‘bad eyes’ that may appear vague to a modern viewer, were attempting to provide specifics. A ‘sore eye’ may feel different than an ‘inflam’d eye’ which may be different still than a ‘diseased eye’—Mathisen interpreted these differentiations as motivated by practitioners desire to differentiate ‘…between minor aggravations and more serious conditions, which might threaten the loss of sight, thereby impacting on the child’s chances of being able to serve an apprenticeship.’^61^ Only one child, 13-year-old Ann Bruce, was noted as being ‘blind from an ulcer’; she was admitted as an inpatient and discharged as cured.

The miscellaneous surgical category displayed the greatest sex disparity overall, with 86.4 per cent male and 13.2 per cent female patients. Overwhelmingly, patients in this category were seen as outpatients (97.4 per cent of cases). The majority (159/228) were male children seen for rupture (i.e., hernia). Buchan notes that ‘Children and very old people are most liable…’ to suffer rupture, children ‘…by excessive crying, coughing, vomiting, or the like.’^62^ Indeed, many of the children from Northampton seen for rupture are noted to have had the condition ‘from birth.’ The number of male children seen for rupture may reflect initial impressions of a case that later turn out to be ‘watery rupture’, or the distention of the scrotum, which Underwood notes is ‘…frequently mistaken by midwives and nurses for a common rupture…’ or retention of the testes, the swelling of which also resembles a hernia.^63^ The majority of the rupture cases are seen as outpatients and resolve as ‘cured.’

The remaining distempers comprised less than five per cent each of the total child visits to Northampton, making any conclusions drawn increasingly tenuous. Neurological issues included distempers such as palsy, convulsions, epileptic fits, and paralysis. Interpretations of neurological patients suffering from epilepsy discharged as ‘cured’ have been questioned by Williams and Sharma, who posit that the term in this case may simply mean seizure-free.^64^ The Miscellaneous medical category included accidents such as swallowing a half penny, and many vague notations of ‘complication.’ Tumours included any children noted as having cancerous or scirrhous growths, as well as general tumours appearing in or on their faces, mouths, lips, cheeks, necks, armpits, legs, groins, and backs, developing over weeks or months. Respiratory issues included asthma, pleurisy, and diagnoses of consumption. The least populous categories were circulatory issues, including dropsy, ascites, and hydrocele, and genitourinary issues, including incontinence, bladder stones, and paraphymosis.

## Embodiment and Northampton

While treatment for children was not the key aim of the voluntary hospital system, Northampton, like many other voluntary institutions, accepted and treated children as inpatients and outpatients. The bodies of these children, inscribed in the existing admission records, are themselves archives of experience and exposure, whether or not we are able to access their experiences from the textual record. Examining the patterning of distempers within and between patients provides insight into the social bodies of eighteenth-century children through the lens of their hospital admissions, their parents, and their institutional recommenders.

During the eighteenth century, the domestic sphere remained the primary environment in which lower-class children received medical care.^65^ Fears of miasma led to recommendations regarding the relationship of fresh air to health, and contemporary commentators despairing over the suffocating slum living conditions of many of the English working class.^66^ The role of diet in children’s health was recognised by figures such as William Cadogan, who wrote to the London Foundling Hospital governors with a list of dietary recommendations, including bread, fresh butter, meats, and fruits depending upon the child’s age.^67^ Analysis of menus from the Northampton Infirmary indicates that inpatients were offered nutritionally adequate sustenance, which was likely ‘…more substantial than that which the local poor could ordinarily afford.’^68^ Levene and colleagues note the critical nature of context for understanding medical admission records, stating that ‘…local industry, epidemiology, or environment clearly cannot give us the whole story, at least for children.’^69^ This statement underscores the importance of the NIECCAD for investigations of children as both individuals and as a collective.

Despite the domestic sphere remaining the primary location for children to receive healthcare, eighteenth-century physicians demonstrated a keen interest in children’s health. Ashley Mathisen asserts that there was at first ‘…a degree of self-consciousness among those practitioners who treated children…’ as the hegemonic view placed authority over children’s health with their mothers, with nurses and midwives closely associated.^70^ Armstrong addresses this belief head-on, stating that ‘…it has been long a common saying in this country, that the best doctor for a child, is an old woman.’^71^ Cadogan asserts that ‘…this Business has been too long fatally left to the Management of Women…’ with Underwood more gently suggesting that ‘The laudable affection of the fondest mother frequently becomes a source of manifold injury to her tender offspring….’^72^ Armstrong admits that he was also previously reticent to treat children, but found that the “…most plausible excuse for declining to practise amongst infants is, that they are not capable of telling their ailments…’ but insists that ‘…the very symptoms themselves will, for the most part, speak for them….’^73^ Buchan echoes this opinion and includes a reference to collaborative care for children, noting ‘It is true, they cannot tell their complaints; but the causes of them may be pretty certainly discovered by observing the symptoms, and putting proper questions to the nurses.’^74^ While it is rare to have the actual words or moods of children recorded, such as nine-year-old Henry Davis, who despite sporting a head wound caused by a falling chimney brick requiring treatment at St. George’s Hospital in London, ‘…told the Nurse cheerfully that his head did not acke’,^75^ the very presence of children as inpatients in hospitals such as Northampton speaks to the willingness of physicians to be part of their care, even if ‘…they cannot, or perhaps will not, tell us their ailments’.^76^

For instance, the database allows for the investigation of embodiment in reference to amputations in children, an irreversible and highly emotive procedure. The first recorded amputation was Edward Allibone, aged thirteen, who was admitted as an inpatient on 31 January 1747 for ‘Distorted Ankle an [sic] amputation.’ The second was published in 1779 in *Medical and Physiological Commentaries*, authored by Mr William Kerr, Senior Physician and Surgeon at Northampton, in an article entitled ‘An Account of the Operation of amputating the Thigh at the upper Articulation.’^77^ Kerr describes in detail the procedure of hip disarticulation he undertook on an unnamed twelve-year-old girl from Kettering, Northamptonshire. The operation greatly relieved her suffering, and the wound apparently healed well, but she died eighteen days following the surgery. A postmortem found widespread lung damage, likely due to tuberculosis, and a psoas abscess which had drained into the area of the amputated joint. The publication of this case report is a milestone in the history of paediatrics, but it was previously disconnected from the individual who underwent the procedure. The unnamed girl has been identified as Sarah Harris through analysis of the NIECCAD.^78^ Sarah’s humanity, life, and suffering are finally reunited with her name. Despite this reunion, there is no textual record of Sarah’s own thoughts on her experience, or how her parents felt about delivering their suffering child to the Northampton General Infirmary for the invasive surgery. While each child’s experience is their own, Sarah’s likely fear and pain may have echoed in the ‘…long…cries expressive of the most horrible pain’ uttered by fifteen-year-old Edward [Quinton] when admitted to St. George’s Hospital, in London, following an eventually fatal fall.^79^ Every child’s humanity and suffering are worthy of attention, despite few of their individual voices having survived.

The percentage of total children’s admissions for all distempers was charted (1744–1773), and in most cases the trendline showed a weak or no relationship.^80^ A slight weak downward trend was present over time for skin disease and miscellaneous medical distemper admissions. These results are valuable as they illustrate that over a thirty-year period, the hospital was steadily providing general care, as was the founders’ aim. While the stated aims of the hospital championed the treatment of acute conditions, many chronic issues were treated. The skin disease and surgical infection distempers included many long-standing cases, some of several years’ duration before presentation at the hospital.

Embodied environmental and exposure risks could include factors such as overcrowded housing and poor-quality diet, while behavioural or situational risks might include occupational tasks and risk-taking behaviours. Though the overall number of children assessed as in- and outpatients was fifty-six per cent male and 43.3 per cent female, an important finding in the distemper results is the relative sex parity for the most populous distempers. Scholars such as Hannah Newton have noted that sex and gender have not yet been explored fully in reference to the medical treatment of children, whereas medical narratives regarding adults more clearly integrate gender differentiation.^81^ Seventeenth-century medical texts suggested that sex had relatively little effect on children’s diseases, with the exception of diseases specifically affecting the genitalia, which may explain the male preponderance of diagnoses of ‘rupture.’^82^ Poverty’s relationship with exposure to poor sanitation, overcrowded housing, and pollution, overlapping with potential undernutrition and early labour, would have affected all children in a household. Age at weaning decreased in Britain over the eighteenth century to 7.25 months, with the rise of artificial feeding and beliefs that colostrum was dangerous.^83^ The introduction of nutrient-poor pap (flour or breadcrumbs in milk or water) could result in general undernutrition and vitamin deficiencies like scurvy.^84^ Further, milk (and the air in overcrowded lodgings) could be infected with tuberculosis.^85^ The relatively equal proportions of male and female children suffering from surgical infection, skin disease, infectious disease, and digestive disease provide clues to environmental risks facing children in Northampton and surrounding areas, and parental investment in their recovery, regardless of their sex or gender.

The sexual division in distempers between male and female children arises in reference to musculoskeletal and trauma distempers, suggestive of a difference in behaviour and potentially gendered household or occupational tasks. The sex-based difference in trauma aligns with previous studies of injuries and accidents in eighteenth-century children, in which the divergence between male and female children is clear and male children are more likely overall to be treated for trauma in a voluntary hospital.^86^ Craig Spence’s exploration of accidents in Early Modern London notes that children have ‘…limited abilities in judging distance and speed…’ which could make them vulnerable to trauma in general.^87^ Levene and colleagues have proposed that this sexual division may relate to ‘…different risks of injury and accident in their daily work and play…’, noting also that parents may ‘…[distinguish] between sons and daughters when seeking medical assistance.’^88^ Indeed, children’s labour may have contributed to their morbidity and trauma patterns. Explorations of child labour during the Industrial Revolution have discussed the value placed on children as a source of potential capital for their families.^89^ While both contemporary observers and historians have found that mortality rates among urban working children were not significantly worse than their rural counterparts, this does not indicate that work was without occupational risks.^90^ Children starting apprenticeship work outside the home would have been at increased risk of acquiring occupational diseases, suffering industrial accidents, and catching infectious distempers.^91^ While the majority of trauma cases do not include details of the cause, making observations regarding gendered activities necessarily tentative, it is worth noting that all five cases in which children were kicked by or fell from horses were boys (Richard Harris, age twelve, 1748; Thomas Sharman, age eleven, 1762; Samuel Cumberpatch, age eight, 1762; Samuel Westley, age eight, 1763; Edward Smith, age eight, 1771). In contrast, children assessed for burns and scalds (without a cause listed) were primarily female (14/20), potentially suggesting domestic work involving fire. Interestingly, four children were seen as outpatients for burns from gunpowder: three boys and one girl. Two of the boys, John Cotton (age twelve) and Thomas Chapman (age thirteen), were both assessed on 11 June 1768, one after the other (as indicated by their outpatient numbers). Whether this was the result of apprenticeship work, domestic duties, or summer mischief is impossible to assess.

Children do not, of course, admit themselves to the hospital. Despite the richness of the admission records, the children are still accessed indirectly, viewed through an institutional lens. Implied in each line of the admission register containing a child are the actions and presence of at least one parent or guardian. Children may or may not have been able to express their symptoms or describe their afflictions, emphasising the importance of parents, particularly the mother, in communicating the child’s distress.^92^ The expansive historiography of childhood in modernity has explored historical parental affection, critiquing Philip Ariès’s assertion of childhood’s insignificance.^93^ Conceptualising the family and children’s place within it requires an exploration of social roles, choices, and sources. Our understanding of the past is driven by the available sources; elite children were more likely to be recorded and thus have formed the foundation of previous understandings of parental-child relations.^94^

One can easily imagine, then and now, both the fear and relief of the parents upon securing inpatient admittance for their children, as well as the difficulty of seeing one’s children endure treatments or lengthy inpatient stays. Newton describes the time and financial resources utilised for at-home medical treatments reported by parents, and writings of grief prior to and at the time of a child’s death.^95^ Health was a common topic in eighteenth-century correspondence, with parents expressing extreme worries regarding their children’s fates. For instance, Elizabeth Wilson, writing to her sister Rebekah Bateman in 1795, states that her son has been unwell ‘…which alarmed my fears…’^96^ while Rebekah’s friend Mary Jane Hodson writes while her daughter is unwell with chickenpox ‘…what I have suffered on her Acc.t since I can’t make you conceive…to see her so afflicted you must supposed how I must be affected…’.^97^ Robert Augustus Johnson writes to his brother George in the 1780s when his daughter Harriet is unwell with fever, of his wife’s response, saying ‘…Mrs Johnson is so much affected with seeing her ill that she stands almost as much in need of assistance as Harriet herself.’^98^ In another case, a child’s health over the course of several years could warrant a letter—Judith Madan, writing to her husband Martin Madan in 1728, worries about their son’s upset stomach, saying ‘…I cannot express my concern & Dread for him…’.^99^ The boy recovers, but falls ill eight years later, with Martin writing to his wife upon their son’s recent recovery with poetic relief that ‘…our Dear little Martin is restor’d to us, & rescued, by the Almighty Power, from the Jaws of Death, the Remembrance of which, I shall endeavour never to forget, but shew my acknowlegments by Praises & Thanksgivings to Good works.’^100^ While not every parent was literate and therefore able to record such emotive words, the intensity of their worry resonates clearly into the present.

Similar sentiments from non-elite families in Northampton can be found between the lines of the admission record, particularly the column recording how long a child had been suffering from a distemper prior to admission. The first Northampton inpatient, thirteen-year-old Thomasin Grace, is recorded as suffering from ‘scald head’ (ringworm) from her infancy—a period of well over a decade. The treatment of infectious disease at Northampton is particularly revelatory. In at least ten cases, sets of two or three siblings are admitted to the infirmary with infectious distempers (e.g., fever, intermittent fever, ague) on the same day. A striking example is the admittance of John (age eight) and Elizabeth (age eleven) Cartwright alongside Sarah (age nine) and James (age twelve) Mann on 10 November 1770. All four are from Northampton and are recommended by the hospital committee, having been ill for between eight to twelve days. These four remained in the hospital until 1 December 1771. Similar situations were recorded at the Bamburgh Castle Dispensary, where sets of siblings, or sometimes larger family groups including parents, are admitted at once suffering from infectious distempers.^101^

The NIECCAD provides value beyond a view into individual children’s afflictions; each line in the register potentially represents conversations between caregivers in non-elite households and reveals networks of relationships and charity in the form of recommenders. Such connections resonate within Foucault’s concept of biopolitics: how social and political power is operationalised through control of people’s bodies. The inter-class connection between health and power is a key element of studying voluntary hospitals, the majority of which were dependent upon charity. Recommenders were recorded for most patients from the opening of the infirmary until early 1774. During this time span, 1706 child patients were recommended, 1448 of whom have their recommenders listed. The most common individual recommender of children was the Hospital Committee itself, which recommended a total of 474 patients, eighty-three of whom were children. Parishes or a parish officer were listed as the recommender in 102 cases. A significant case is that of a four-year-old inpatient named Stephen Peasnall, admitted for dysentery and recommended by the parish of Bugbrooke. This infectious patient was accepted as an inpatient, in contravention of both the age and non-infectious rules. There are fewer than ten total non-surgical inpatients under seven years old out of the hundreds listed, which directly defies the hospital rules under the statutes of the hospital.^102^ The ability of a parish to recommend a prohibited inpatient may be a testament to the power of the parish itself, or otherwise denote a culture of care that occasionally performed outside of policy.

Individual hospital subscribers generally only recommended a handful of patients, making individuals like Ann Isted and founder Dr Doddridge’s contributions of note. Ann Isted recommended at least eighteen child patients between 1744 to 1762.^103^ She recommended child patients from infancy until the early teenage years, although only five of these patients, all outpatients, were under seven years of age. Ann Isted’s connections to the community appear evident through her recommendation of two children from the same parish, recommended on the same day: Robert (age seven) and Susan (age eleven) Watts, both brought low by fever, which they had suffered for a month. Both were treated as outpatients, deemed cured, and returned to Hardingston. Dr Doddridge, one of the founding members of the hospital, was another prolific recommender of children. A total of twelve child recommendations mention him, including one under seven. For instance, Doddridge recommended eight-year-old Elizabeth Ager as an inpatient against regulations with a one-month history of fever. Doddridge had lost a daughter, Elizabeth, aged five years, due to consumption in Northampton in 1736, as well as a son, Samuel, who died in infancy. In 1737, he published a sermon on child bereavement, expressing how Elizabeth was ‘…able to give me a Degree of Delight, and consequently of Distress, which I did not before think it possible…’.^104^

Both are significant as they show that child healthcare through the Northampton General Infirmary was directly supported through its practices, even by the individuals responsible for treating patients. Though a full consideration of the topic is beyond the scope of this manuscript, it is worthy of note that the Isteds (Ann and her nephew, Ambrose Isted, another frequent recommender) were absentee plantation owners.^105^ That an injured child in Northampton, UK, could potentially be sponsored through the donation of funds made from the labour of enslaved peoples in Jamaica expands our understanding of the complex networks of risk possible to uncover through resources such as the NIECCAD.

## Conclusion

The admittance of Thomasin Grace, a thirteen-year-old girl with longstanding ringworm, as the first inpatient to the Northampton General Infirmary symbolically places children and their future care as a foundational part of the story of the then-nascent institution.^106^ This research demonstrates that care for children continued from this day; while patient ages were no longer recorded after 1804, it is reasonable to assume that children were still treated and admitted by the infirmary. To quantify this further, however, would require individual cases to be identified and linked with a high degree of certainty to local parish records. One hint that child admissions continued after the period under study is the presence of a rare copy of Dr John Darwall’s *Plain Instructions for the Management of Infants, with Practical Observations on the Disorders Incident to Children*, published in 1830, in the Northampton General Hospital’s library and archives.^107^ Indeed, the institution itself, still operating today and known as the Northampton General Hospital since 1905, has continued to expand the range of child health services offered to the local population. Notable examples include a children’s ward opened at the Northampton Infirmary in 1889, a premature baby unit opened in 1950 (moved to the Gosset Neonatal Unit in 1965), a Child Development Centre opened in 1974, and a Paediatric Emergency Department in 2021.^108^

The treatment of working-class individuals in general hospitals as a means of returning working folk to labour has been highlighted in studies of admission records.^109^ This institutional exchange—health for labour—resonates within the concept of biopolitics, and scholars such as Benzaquén have explored how the increased medical attention paid toward children in the eighteenth century ‘…for the child’s own sake, for the sake of the family and for the sake of the state.’^110^ While future labour is an important aspect of the treatment of children, broader aspects of their lives and experiences should not be sidelined. For instance, the infirmary’s first patient, Thomasin Grace, spent 101 days in hospital being treated for a case of ‘scald head’ (ringworm) and was discharged ‘cured’. A Thomasin Grace appears again eighteen years later in Shoreditch, London, signing her marriage certificate on 17 May 1762.^111^ Assuming these Thomasins are one and the same, might she have been instructed in reading and writing during her original 1744 admission as per Infirmary statutes? Whether that was indeed the case or not, Thomasin’s admission highlights that children may have been treated at the Northampton Infirmary so they could labour, but also in the hopes that they would have the opportunity to grow up.

Decisions to allow children into the hospital were not arbitrary, and the institution clearly did not rely on a single voice and set of values to guide its care. Understanding the network of care underscores the impact of community and inter-class relationships, which persisted despite such stark divides of power. Following the money brings a harsh reality into view, one in which enslavement and forced labour of people in a nation across the ocean potentially funds children’s care sponsorship in another. Such possible connections complicate the concept of risk embodiment, as child patients embody the risks of their own environment and are relieved by the risks forced upon others. Future work built from the foundation of the NIECCAD will further our understanding of children’s place in the community, as their value—emotional and financial—to their families is clearly communicated in the register. Beyond this, our understanding of the Northampton Infirmary’s role in the Early Modern East Midlands medical marketplace will benefit from the experiences recorded in the NIECCAD.

## Footnotes

1

Fielding H. Garrison , ‘History of Pediatrics’, in Isaac A. Abt (ed), Abt-Garrison History of Pediatrics (London: WB Saunders, 1965), 1.

2

William Cadogan , An Essay Upon Nursing, and the Management of Children, From Their Birth to Three Years of Age (London: Printed for J. Roberts, 1748); William Buchan , Domestic Medicine; or, A Treatise on the Prevention and Cure of Diseases By Regimen and Simple Medicines (Edinburgh: Balfour, Auld, and Smellie, 1769), page references in forthcoming notes are drawn from the third edition (London: Printed for W. Strahan; T. Cadell in the Strand; and J. Balfour, and W. Creech, at Edinburgh, 1774); George Armstrong , An Essay on the Diseases Most Fatal to Infants. To Which are Added Rules to be Observed in the Nursing of Children: With a Particular View to Those Who are Brought Up by Hand (London: Printed for T. Cadell, 1767); Michael Underwood , A Treatise on the Diseases of Children, With General Directions for the Management of Infants From The Birth (London: Printed for J. Mathews, 1784), page references in forthcoming notes are drawn from the 1793 edition (Philadelphia: Printed by T. Dodson, 1793); See also Claire Marie Rennie, ‘The Care of Sick Children in Eighteenth-Century England’ (unpublished PhD thesis: University of Leeds, 2016).

3

Thomas Phaer , The Boke of Chyldren (London: Edward Whitechurch, 1544).

4

Hannah Newton , ‘Children’s Physic: Medical Perceptions and Treatments of Sick Children in Early Modern England, c. 1580-1720’, Social History of Medicine, 23, 3 (2010), 456–74, 45626306061 10.1093/shm/hkq006PMC4545517; Hannah Newton , The Sick Child in Early Modern England, 1580-1720 (Oxford: Oxford University Press, 2012); Rennie, *op. cit* (note 2), 16.

5

P.M. Dunn , ‘George Armstrong MD (1719-1789) and his Dispensary for the Infant Poor’, Archives of the Diseases of Childhood, 87 (2002), 228–31, 23010.1136/fn.87.3.F228PMC172147012391002.

6

Andrew N. Williams , ‘Eighteenth-century Child Health Care in a Northampton Infirmary: a Provincial English Hospital’, Family & Community History, 10, 2 (2007), 153–66, 155; Alysa Levene , Jonathan Reinarz and Andrew N. Williams , ‘Child Patients and Voluntary Hospitals in Eighteenth-Century England’, Family & Community History, 15, 1 (2012), 15–33, 20; Madeleine Mant , ‘Children in the London: Inpatient Care in a Voluntary General Hospital’, Medical History, 62, 3 (2018), 295–313, 30029886865 10.1017/mdh.2018.24PMC6113752.

7

Roy Porter , Enlightenment. Britain and the Creation of the Modern (London: World Penguin Books, 2001), 206–7; Lindsay Granshaw , ‘The hospital’ in W.F. Bynum and Roy Porter (eds.), Companion Encyclopedia of the History of Medicine (London: Polity, 1993), 1180–203, 1185.

8

Roy Porter , ‘The Patient’s View: Doing Medical History from Below’, Theory and Society, 14, 2 (1985): 175–98, 182–311618212 .

9 Williams, *op. cit.* (note 6), 155.

10

County Hospital at Northampton for Sick and Lame Poor , Statutes Rules and Orders for the Government of the County Hospital for Sick and Lame Poor, Establish’d in the Town of Northampton (Northampton: Printed by William Dicey, 1743), 22 .

11 Levene et al., *op. cit.* (note 6), 17.

12 Levene et al., *op. cit.* (note 6), 31.

13 Levene et al., *op. cit.* (note 6), 19.

14

Andrew N. Williams and Raman M. Sharma , ‘Children in Hospitals Before There Were Children’s Hospitals’, Pediatrics, 134, 3 (2014), 425–7, 42625113297 10.1542/peds.2013-0746.

15

Claire Phillips , ‘Medical Care for Working-Class Children in the Late Eighteenth and Early Nineteenth Century’ in Oliver Betts , Laura Harrison , and Laura Christine Price (eds.) Doing Working-Class History: Research, Heritage, and Engagement (London: Routledge, 2024): 149–61, 150.

16

Alun Withey , ‘Medicine and Charity in Eighteenth-century Northumberland: The Early Years of the Bamburgh Castle Dispensary and Surgery, c. 1772-1802’, Social History of Medicine 29, 3 (2016), 467–89, 48427482145 10.1093/shm/hkw008PMC4966483.

17
*Ibid.*, 472; see also Jonathan Reinarz and Leonard Schwarz , ‘Introduction’, in Jonathan Reinarz and Leonard Schwarz (eds.), Medicine and the Workhouse (Rochester: University of Rochester Press, 2013), 3–5 .

18

Andrew N. Williams , ‘A Vaccine Scare in 19^th^ century Northampton’, Archives of Disease in Childhood 90 (2005), 1204 10.1136/adc.2005.081034PMC172017116243891.

19

Margaret Pelling , ‘Child Health as a Social Value’, Social History of Medicine, 1, 2 (1988), 135–64, 16311621945 10.1093/shm/1.2.135.

20

Sidney Lee , ed., Dictionary of National Biography, Vol. 54 (London: Smith, Elder & Co., 1898), 417–18.

21

Francis F. Waddy , A History of Northampton General Hospital 1743 to 1948 (Northampton: The Guildhall Press, 1974), 4 .

22

Malcolm Deacon , Philip Doddridge of Northampton 1702-1751 (Northampton: Northamptonshire Libraries, 1980), 105 .

23 Lee, *op cit.* (note 20), 417–18.

24

Philip Doddridge , ‘Compassion to the Sick recommended and urged, in a Sermon preached at Northampton, September 4, 1743. In Favour of a Design then opening to erect a County Infirmary there for the Relief of the Poor Sick and Lame’ (London: M. Fenner and W. Dicey, 1743), 2 .

25
*Ibid.*, ix.

26 County Hospital at Northampton for Sick and Lame Poor, *op. cit.* (note 10), 22.

27

Andrew N. Williams , ’The Joy to Bless and To Relieve Mankind’: Child Healthcare at Northampton General Infirmary 1744’, Archives of Diseases in Childhood, 90 (2005), 1227–9, 122716301548 10.1136/adc.2005.073411PMC1720238.

28

Richard Grey , The Encouragement to Works of Charity and Mercy, from Christ’s Acceptance of them as done to Himself. A Sermon Preached in the Parish Church of All-Saints in Northampton, Before the President and Governors of the County Infirmary for Sick and Lame Poor, on Thursday, March 29, 1744 (Northampton: W Dicey, 1744), 20 .

29 County Hospital at Northampton for Sick and Lame Poor, *op. cit.* (note 10), 22.

30 James Stonhouse, *Friendly Advice to a Patient; Calculated more particularly for the Use of the* Sick, *belonging to the* Infirmaries*, as well the* Out-Patients*, as those* within the House; *tho’ the greatest Part of it is suitable and of equal Service to every sick person* (London: Printed by John Rivington, for J.F and C. Rivington, Booksellers, No. 62 in St. Paul’s Church-Yard, 1781), 3.

31

Andrew N. Williams , ‘Four Candles. Original Perspectives and Insights into 18^th^ Century Hospital Child Healthcare’, Archives of Diseases in Childhood, 92 (2007), 75–9, 7517185447 10.1136/adc.2006.095729PMC2083136.

32 State of the County Hospital in Northampton 1744. Northampton Infirmary Archives NGH/1/6/1 1743–1826.

33 State of the County Hospital in Northampton 1745. Northampton Infirmary Archives NGH/1/6/1 1743–1826.

34 Waddy, *op. cit.* (note 21), 17.

35
*Ibid.*, 26–30.

36
*Ibid.*, 175.

37 Following the age categories of Levene et al., *op. cit.* (note 6), 19 and Mant, *op. cit.* (note 6), 300.

38 County Hospital at Northampton for Sick and Lame Poor, *op. cit.* (note 10), 22. For a European example see Signild Vallgarda , ‘Who Went to a General Hospital in the Eighteenth and Nineteenth Centuries in Copenhagen?’, European Journal of Public Health, 9, 2 (1999), 97–102, 99.

39 Levene et al., *op. cit.* (note 6), 19.

40

Guenter Risse , Hospital Life in Enlightenment Scotland: Care and Teaching at the Royal Infirmary of Edinburgh (Cambridge: Cambridge University Press, 1986), 124 .

41 Ashley Mathisen, ‘Treating the Children of the Poor: Institutions and the Construction of Medical Authority in Eighteenth-century London’ (unpublished PhD thesis: University of Oxford, 2011), 171, 179, 181.

42 Mant, *op. cit.* (note 6), 302.

43 S. Cherry, ‘The Hospital and Population Growth: Part 1 The Voluntary General Hospitals, Mortality and Local Populations in the English Provinces in the Eighteenth and Nineteenth Centuries, *Population Studies*, 34, 1 (1980), 59–75, 62.

44 Buchan, *op. cit.* (note 2), 624.

45
*Ibid.*, 624.

46 Levene et al., *op. cit.* (note 6), 22.

47 Underwood, *op. cit.* (note 2), 141, 142.

48

Mary J. Dobson , Contours of Death and Disease in Early Modern England (Cambridge, Cambridge University Press, 1997), 237, 240.

49 Buchan, *op. cit.* (note 2), 28.

50

C. Booth , John Haygarth, FRS (1740-1827): a Physician of the Enlightenment (Philadelphia: American Philosophical Society, 2005), 64 .

51

William Buchan , Domestic medicine: or, A treatise on the prevention and cure of disease by regimen and simple medicines; With an appendix containing a dispensatory. For the use of private practitioners (London: Printed for W. Strahan, T. Cadell, J. Balfour, and W. Creech, 1774), 604–5.

52

Alysa Levene and Kevin Siena , ‘Reporting Dirt and Disease: Child Ill-Health in Seventeenth- and Eighteenth-century England’, J*ournal of Literature and Science* , 6, 1 (2013), 1–17, 8; Kevin Siena , ‘The Moral Biology of “The Itch” in Eighteenth-Century Britain’ in Jonathan Reinarz and Kevin Siena (eds.) A Medical History of Skin: Scratching the Surface (London: Pickering & Chatto, 2013), 71–84, 71 .

53

Thomas Spooner , Short Account of the Itch (London: J. Roberts, and A. Dodd, 1728).

54
*Ibid.*, 604.

55 Levene et al., *op. cit.* (note 6), 22.

56 Mathisen thesis, *op. cit.* (note 41), 176.

57 Cherry, *op. cit.* (note 43), 70–1.

58 Buchan, *op. cit.* (note 2), 397, 399, 402.

59

Ashley Mathisen , ‘“So that they may be usefull to themselves and the community”: Charting Childhood Disability in an Eighteenth-Century Institution’, Journal of the History of Childhood and Youth, 8, 2 (2015), 191–210, 194.

60 Underwood, *op. cit.* (note 2), 229–35.

61 Mathisen, *op. cit.* (note 41), 188.

62 Buchan, *op. cit.* (note 2), 648.

63 Underwood, *op. cit.* (note 2), 280–4.

64 Williams and Sharma, *op. cit.* (note 14), 426.

65

Alun Withey , Physick and the Family: Health, Medicine and Care in Wales, 1600-1750 (Manchester, Manchester University Press, 2011), especially Chapter 5 “Care and the Welsh medical home” 99–120; Rennie, *op. cit.* (note 2), 1.

66 Buchan, *op. cit.* (note 2), 33–5; Margaret Pelling , “Skirting the City? Disease, Social Change and Divided Households in the Seventeenth Century,’ in Paul Griffiths and Mark S.R. Jenner (eds.), Londinopolis (Manchester: Manchester University Press, 2000), 154–75, 156–8.

67 Cadogan, *Essay Upon Nursing*, 30.

68

G. Denny , P. Sundvall , S.J. Thornton , Jonathan Reinarz , and Andrew N. Williams , ‘Historical and Contemporary Perspectives on Children’s Diets: Is Choice Always in the Patients’ Best Interest?’ Medical Humanities, 36, 1 (2010), 14–8, 1621393268 10.1136/jmh.2009.003368.

69 Levene et al., *op. cit.* (note 6), 23.

70

Ashley Mathisen , ‘Mineral Waters, Electricity, and Hemlock: Devising Therapeutics for Children in Eighteenth-Century Institutions’, Medical History 57, 1 (2013), 28–44, 4323393401 10.1017/mdh.2012.79PMC3566754.

71 Armstrong, *op. cit.* (note 2), 3.

72 Cadogan, *op. cit.* (note 2), 3; Underwood, *op. cit.* (note 2), 2.

73 Armstrong, *op. cit.* (note 2), 4, 6.

74 Buchan, *op. cit.* (note 2), 7.

75 Royal College of Surgeons (RCS) MS0470/3/1 62 Surgical Cases and Commentaries, 1820–60, 55–7.

76 Armstrong, *op. cit.* (note 2), 10.

77 William Kerr, ‘An Account of the Operation of amputating the Thigh at the upper Articulation, lately performed by Mr. William Kerr, Surgeon to the Royal Regiment of Horse-Guards Blue, and to the Hospital in Northampton. Communicated to Dr Duncan, by Dr Toll, surgeon to the Fourth Regiment of Dragoons. *Medical and Physiological Commentaries*, 6 (1779), 337–42.

78

Ramanathan Natarajan , Fred O’Dell , Madeleine Mant , and Andrew Williams , ‘18th-Century Amputations; Two Case Reports’, Archives of Disease in Childhood, 110, 5 (2025), 405–639904569 10.1136/archdischild-2024-327819.

79 Royal College of Surgeons (RCS) MS0470/3/1 62 Surgical Cases and Commentaries, 1820–60, 40–2.

80 R2 values: Surgical infection (0.038); Infectious disease (0.164); Skin disease (0.231), slight downward trend; Musculoskeletal (0.006); Digestive system (0.002); Trauma (0.023); Eye problems (0.002); Miscellaneous surgical (0); Neurological (0.002); Miscellaneous medical (0.232), slight downward trend; Tumours, cancers, growths (0.129); Respiratory disease (0.01); Genitourinary (0.056); Circulatory (0.027)

81 Newton, *op. cit.* (note 4), 460.

82
*Ibid.*, 461.

83

Valerie A. Fildes , Breasts, Bottles, and Babies: A History of Infant Feeding (Edinburgh: Edinburgh University Press, 1986), 81, 370.

84
*Ibid.*, 213.

85

Margaret Cox , Life and Death in Spitalfields 1700-1850 (York: Council for British Archaeology, 1996), 55 .

86 Mant, *op. cit.* (note 6), 302, 305; Barbara A. Hanawalt , ‘Childrearing Among the Lower Classes of Late Medieval England’, Journal of Interdisciplinary History, 8 (1977), 1–22, 16, 1911632277 .

87 Craig Spence, *Accident and Violent Death in Early Modern London 1650-1750*, Studies in Early Modern Cultural, Political and Social History, Vol. 25 (Woodbridge: Boydell, 2016), 120.

88 Levene et al., *op. cit.* (note 6), 20.

89

Peter Kirby , Child Workers and Industrial Health in Britain, 1780-1850 (Woodbridge: The Boydell Press, 2013), 39–46 ; Jane Humphries , Childhood and Child Labour in the British Industrial Revolution (Cambridge: Cambridge University Press, 2010).

90 Kirby, *op. cit.* (note 89), 46–7.

91 Pelling, *op. cit.* (note 19), 156, 161.

92

Iris Ritzmann , ‘Children as Patients in German Speaking Regions in the Eighteenth-Century’, in Anja Müller (ed.), Fashioning Childhood in the Eighteenth Century: Age and Identity (Aldershot: Ashgate, 2006), 25–32 .

93

Philip Ariès , Centuries of Childhood: A Social History of Family Life, Robert Baldick (trans.), (New York: Alfred A. Knopf, 1962); Steven Ozment , When Fathers Ruled: Family Life in Reformation Europe (Cambridge, Mass: Harvard University Press, 1983); Linda Pollock , Forgotten Children: Parent-Child Relations from 1500-1900 (Cambridge: Cambridge University Press, 1983); Ralph Houlbrooke , The English Family: 1450-1700 (London: Routledge, 1984); Hugh Cunningham , ‘Histories of Childhood’, American Historical Review, 103, 4 (1998), 1195–208.

94 Mathisen, *op. cit.* (note 41), 13; Stephen Wilson , ‘The Myth of Motherhood a Myth: The Historical View of European Child-Rearing’, Social History, 9, 2 (1984), 181–98, 185; S. Ryan Johansson , ‘Centuries of Childhood/Centuries of Parenting: Philippe Ariès and the Modernization of Privileged Infancy’, Journal of Family History, 12, 4 (1987), 343–65.

95

Hannah Newton , ‘The Sick Child in Early Modern England, 1580-1720,’ Endeavour, 38, 2 (2014), 122–9, 12624874090 10.1016/j.endeavour.2014.04.001PMC4330552.

96 Elizabeth Wilson to Rebekah Bateman, 1 December 1795, 1121795: Beinecke Rare Book and Manuscript Library, Yale University, Bateman Family Papers, OSB MSS 32 Box 2, Folder 36 (42).

97 Mary Jane Hodson to Rebekah Bateman, 14 October 1786, 14101786: Beinecke Rare Book and Manuscript Library, Yale University, Bateman Family Papers, OSB MSS 32 Box 2, Folder 33 (2).

98 Robert Augustus Johnson to George William Johnson, 27 June 178?, 276: Bodleian Libraries, University of Oxford, Johnson Family, MS. Don. C. 19398.

99 Judith Madan to Martin Madan, 13 April 1728, 1341728: Bodleian Libraries, University of Oxford, Madan Family, Eng Letter C.284 f.100.

100 Martin Madan to Judith Madan, 6 July 1736, 671736: Bodleian Libraries, University of Oxford, Madan Family, Eng Letter C.284 f.142.

101 Phillips, *op. cit.* (note 15), 155.

102 County Hospital at Northampton for Sick and Lame Poor, *op. cit.* (note 10), 22.

103 Other patients are recommended by a ‘Mrs. A. Isted’ and ‘Mrs. Isted’ which could represent more sponsorships by Anne. One Mrs. Phillippa Isted and Mrs. Mary Isted also appear.

104

P. Doddridge , Submission to Divine Providence in the Death of Children, recommended and inforced, in a Sermon preached at Northampton on the DEATH of a very amiable and hopeful CHILD, about five years old, 2nd ed. (London: Printed for R. Hett, at the Bible and Crown in the Poultry, 1740), i.

105 Profiles for Ann(e) Isted (née Rose), her sister Ann(e) Isted (who is the likely recommender), and her Ambrose Isted are available at Centre for the Study of the Legacies of British Slavery (https://wwwdepts-live.ucl.ac.uk/lbs/estate/view/2604).

106 Williams, *op. cit.* (note 6), 160.

107

J. Darwall , Plain Instructions for the Management of Infants, with Practical Observations on the Disorders Incident to Children (London: Whittaker, Trescher and Arnot, 1830).

108 Waddy, *op. cit.* (note 21), 46, 152; Fred O’Dell and Andrew N. Williams , ‘Dr. Isaac Henry Gosset (1907-1965): Inventor of The “Gosset” Icterometer and founder of Northamptonshire Neonatal Pediatrics’, Archives of Disease in Childhood Fetal & Neonatal Edition, 101, 3 (2016), F264–526251291 10.1136/archdischild-2015-309161; Andrew N. Williams , B. Mold , L. Kilbey , and P. Naganna , ‘Forty Years of Referrals and Outcomes to a UK Child Development Centre (CDC): Has Demand Plateaued?’, Child: Care, Health and Development, 44, 3 (2018), 1–6 29460480 10.1111/cch.12552.

109 Mant, *op. cit.* (note 6), 298, 303, 312.

110

Adriana S. Benzaquén , ‘The Doctor and the Child: Medical Preservation and Management of Children in the Eighteenth Century’, in Anja Müller (ed.), Fashioning Children in the Eighteenth Century: Age and Identity (Aldershot: Ashgate, 2006), 13–24, 24.

111 London, England, Marriages and Banns, 1754-1921. Church of England Parish Registers, 1754–1921. The London Archives, London. Hosted by Ancestry.co.uk: https://www.ancestry.co.uk/discoveryui-content/search/collections/1623/records/1482882

